# Over-expression of growth differentiation factor 15 (GDF15) preventing cold ischemia reperfusion (I/R) injury in heart transplantation through Foxo3a signaling

**DOI:** 10.18632/oncotarget.16607

**Published:** 2017-03-27

**Authors:** Yixin Zhang, Lisa A. Moszczynski, Qing Liu, Jifu Jiang, Duo Zhao, Douglas Quan, Tina Mele, Vivian McAlister, Anthony Jevnikar, Seung Joon Baek, Kexiang Liu, Xiufen Zheng

**Affiliations:** ^1^ Department of Cardiovascular Surgery, The Second Hospital, Jilin University, Changchun, China; ^2^ Department of Pathology, Western University, Ontario, Canada; ^3^ Department of Surgery, Western University, Ontario, Canada; ^4^ Lawson Health Research Institute, Ontario, Canada; ^5^ London Health Sciences Centre, Ontario, Canada; ^6^ Department of Medicine, Western University, Ontario, Canada; ^7^ Matthew Mailing Centre, London Health Sciences Centre, Ontario, Canada; ^8^ Department of Biomedical and Diagnostic Sciences, College of Veterinary Medicine, University of Tennessee, Knoxville, TN, USA

**Keywords:** GDF15, ischemia reperfusion injury, heart transplantation, Foxo3a

## Abstract

Ischemia reperfusion (I/R) injury which inevitably occurs during heart transplantation is the major factor leading to organ failure and graft rejection. In order to develop new therapies to prevent I/R injury, we used both a murine heart transplantation model with 24 hour cold I/R and an *in vitro* cell culture system to determine whether growth differentiation factor 15 (GDF15) is a protective factor in preventing I/R injury in heart transplantation and to further investigate underlying mechanisms of I/R injury. We found that cold I/R caused severe damage to the endocardium, epicardium and myocardium of heart grafts from wild type C57BL/6 mice, whereas grafts from GDF15 transgenic (TG) mice showed less damage as demonstrated by decreased cell apoptosis/death, decreased neutrophils infiltration and the preservation of the normal structure of the heart. Over-expression of GDF15 reduced expression of phosphorylated RelA p65, pre-inflammatory and pro-apoptotic genes while it enhanced Foxo3a phosphorylation *in vitro* and *in vivo*. Over-expression of GDF15 inhibited cell apoptosis/death and reduced neutrophil infiltration. In conclusion, this study, for the first time, demonstrates that GDF15 is a promising target for preventing cold I/R injury in heart transplantation. This study also shows that the resultant protective effects are mediated by the Foxo3 and NFκB signaling pathways.

## INTRODUCTION

Heart disease is the leading cause of death in the United States, causing more than 375,000 deaths yearly [1]. Heart transplantation is a standard treatment for the end stage of heart diseases. However, this treatment is severely impeded by ischemia reperfusion injury which inevitably occurs during donor excision, organ preservation, surgical operation and after surgery. Ultimately, this injury can lead to organ failure and graft rejection.

During ischemia, anaerobic respiration causes the formation of harmful metabolites leading to cell damage and death. Reperfusion further exacerbates this damage through increasing the inflammatory response and increasing apoptosis [2]. Clinics currently place donor hearts in preservation solution at low temperature during transportation and while waiting for tests. This is done to slow metabolic activity, thus reducing anaerobic respiration and its downstream effects, in order to reduce I/R injury and to preserve organ viability. To differentiate from warm I/R injury, the specific setting in which I/R injury occurs involving low temperature storage in organ transplantation has been termed ‘cold I/R’. However, I/R injury remains an unresolved problem clinically, as cold temperature does not completely stop metabolic activity and the consequent injury is amplified during reperfusion. The duration of ischemia correlates with the degree of graft rejection, especially with respect to immunosuppressant-resistant chronic rejection [3]. The risk of primary graft failure and death rises dramatically as ischemic time increases [4]. In cardiac transplantation, cold ischemic storage of human hearts is limited to 4-6 h, which severely reduces the use of donor organs. In recent years, maintaining organ viability has become more challenging because the shortage of donors has led to broader criteria for donor acceptability and consequently to organs with greater compromise [5].

Many attempts including our previous study using small interference RNA (siRNA) have been tried, and limited successes have been reported in animal studies [6]. There is an urgent need for increased understanding of cold IR injury and to find new targets.

Growth Differentiation Factor 15 (GDF15), also known as macrophage inhibitory cytokine 1 [7], placental bone morphogenetic protein [8], placental transforming growth factor [9], prostate derived factor [10] or nonsteroidal anti-inflammatory drug-activated gene 1 (NAG1) is a member of the transforming growth factor beta (TGF-β) superfamily [11]. It is known to have pleiotropic functions involving stress response, inflammation, tumorigenesis, metastasis, angiogenesis, and tissue injury and recovery pathways [12]. It has been reported that GDF15 is upregulated in ischemic heart tissues in ae coronary artery ligation model [13], in rat liver tissues exposed to ischemic preconditioning before ischemia [14] and in the heart in response to hypertrophic conditions [15]. This quick up-regulation of GDF15 is aimed at preventing cells from further damage [13, 14]. A more recent study by Zhang [16], reported that the expression of GDF15 was up-regulated in myocardial tissues after undergoing I/R, and that this up-regulation of GDF15 reached a peak at 24h after reperfusion in a rat descending artery ligation myocardia I/R model with 1h ischemia. Zhang et al demonstrated that GDF15 has a protective effect on warm I/R injury by inhibiting the inflammatory response that predominantly involves neutrophil infiltration and trans-endothelial migration [16]. In addition, it was found that GDF15 was up-regulated in cold I/R injured heart grafts in heart transplantation detected by cDNA microarrays [17].

However, the specific impact of GDF15 on cold I/R in heart transplantation remains unknown and its underlying mechanism is unknown. In this study, we determined that GDF15 has a protective effect on cold I/R in heart transplantation using GDF15 transgenic mice *in vivo* and using GDF15 expression adenovirus *in vitro*. We discovered GDF15 exerts the protective effect through interaction with Foxo3a and NFκB signalling.

## RESULTS

### GDF15 protects the heart from I/R injury in heart transplantation

**Figure 1:**
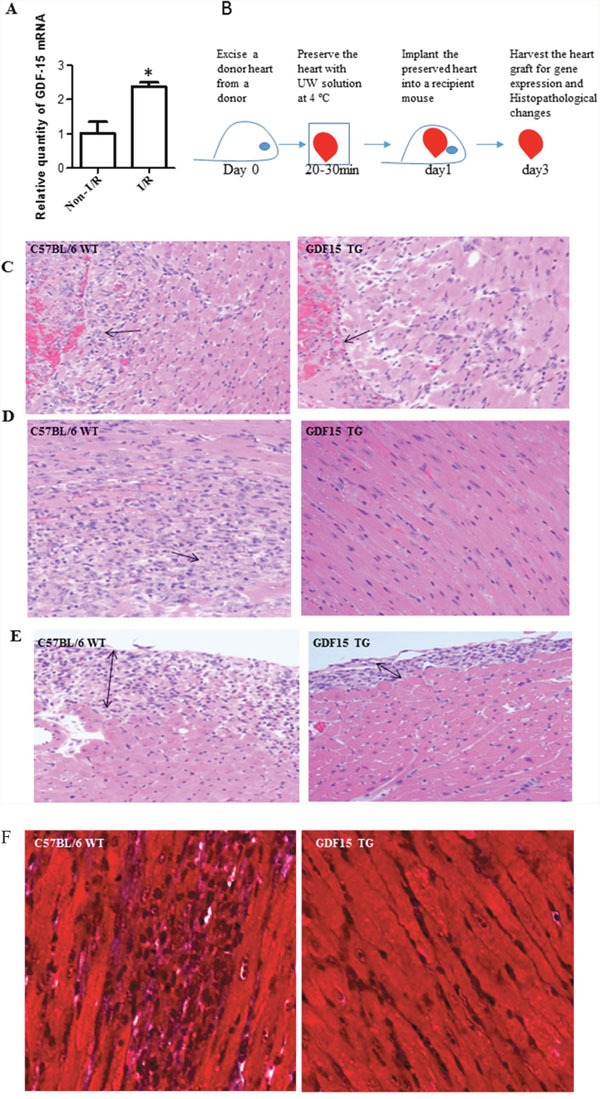
GDF15 protects hearts from I/R injury in heart transplantation **(A)** I/R increased GDF15 expression. Donor hearts were harvested from WT C57BL/6 (n=6) and preserved with UW solution at 4°C for 24 h before implantation into syngeneic recipients. 24 h after transplantation, heart grafts were harvested for gene expression. **(B)** A timeline for heart transplantation. **(C-F)** H&E staining. Donor hearts were harvested from WT C57BL/6 and GDF15 TG mice (n=6, each) and preserved at 4°C for 24 h, followed by a heterotopic syngeneic transplantation. Three days after transplantation, heart grafts were harvested for H&E staining. (C) Endocardium infiltrated with neutrophils. (D) Cardiomyocyte damage. (E) Epicardium thickening. (F) Trichrome staining for fibrosis. Representative images from 6 independent grafts per group. Images were taken at X 200 magnification.

Using a mouse heart transplantation model, our previous study showed that cold I/R caused heart graft damage and up-regulates GDF 15 expression in heart grafts, using cDNA microarray analysis [17]. However, it is unknown whether the up-regulation of GDF15 is causative or protective in response to cold I/R in heart transplantation. In this study, we first validated the expression of GDF15 in cold I/R injured hearts in heart transplantation. We excised donor hearts, preserved donor organs with UW solution for 24 h at 4°C, followed by a syngeneic heterotopic heart transplantation. 24 h later, implanted hearts were harvested for gene expression by qRT-PCR. The result showed that 24 h cold I/R increased the expression of GDF15 2.37±0.225 folds as compared to the control grafts without I/R injury (Figure 1A), which is consistent with our microarray data about up-regulation of GDF15 I/R.

Next, we investigated the impact of GDF15 on cold I/R injury in heart transplantation. We used GDF15 transgenic mice that had C57BL/6 background with over-expression of human GDF15 gene [18]. As laid out in Figure 1B, we retrieved donor hearts from GDF15 transgenic mice and wild type C57BL/6 mice, preserved donor hearts in UW solution for 24h at 4°C and then implanted them into syngeneic mice. The specific cold preservation time of 24 hours was chosen based on our previous cold ischemia time point study. We observed that the transplanted heart from wild type mice did not start to beat after implantation, while the hearts from GDF15 TG mice immediately beat once they were re-vascularized and beat strongly with steady speeds, indicating that GDF15 TG heart grafts had a better function. Three days after transplantation we harvested heart grafts to assess I/R injury by examining histopathological changes with H&E staining. Figure 1C-1E showed the histopathological changes in heart grafts including the endocardium (Figure 1C), myocardium (Figure 1D), and epicardium (Figure 1E). In the wild type grafts, the epicardium was infiltrated by neutrophils and became thick, and both the epicardium and endocardium were full of fibrous tissue. The wild type grafts also showed severe cardiomyocyte damage and fibrosis. Neutrophils also infiltrated in the myocardium. Neutrophil infiltration was confirmed by MPO assays (data not shown). By contrast, grafts from GDF15 TG mice showed less damage as demonstrated by less neutrophil infiltration and preservation of normal heart structure. Myocardial tissue maintained normal structural morphology and cardiomyocyte damage was considerably less severe and only observed in fewer areas. Fibrosis in heart tissues was further confirmed by Trichrome staining (Figure 1F). In order to quantify the injury, we evaluated epicardium, endocardium, infarction, neutrophil infiltration and fibrosis using a five point scoring system as described in the Materials & Methods section. As shown in Table 1, GDF15 TG heart grafts had significantly less injury than wild type heart grafts, indicating that GDF15 is a protective molecule in preventing cold I/R injury in heart transplantation.

**Table 1:** Histopathological change in I/R injured heart grafts

	Epicardium injury	Endocardium injury	Infarction	Neutrophil infiltration	Fibrosis
WT	3.5 ± 0.2887	3.167±0.4410	4 ± 0.2887	3.333±0.333	3.367±0.1856
GDF15TG	2.25 ± 0.3227	2 ± 0.2041	1.875± 0.427	2.375±0.125	1.750±0.1443
*p*	0.0395	0.0457	0.0127	0.0289	0.0009

### GDF15 inhibits cell apoptosis and reduces expression of proinflammatory cytokine production in heart graft *in vivo*

**Figure 2:**
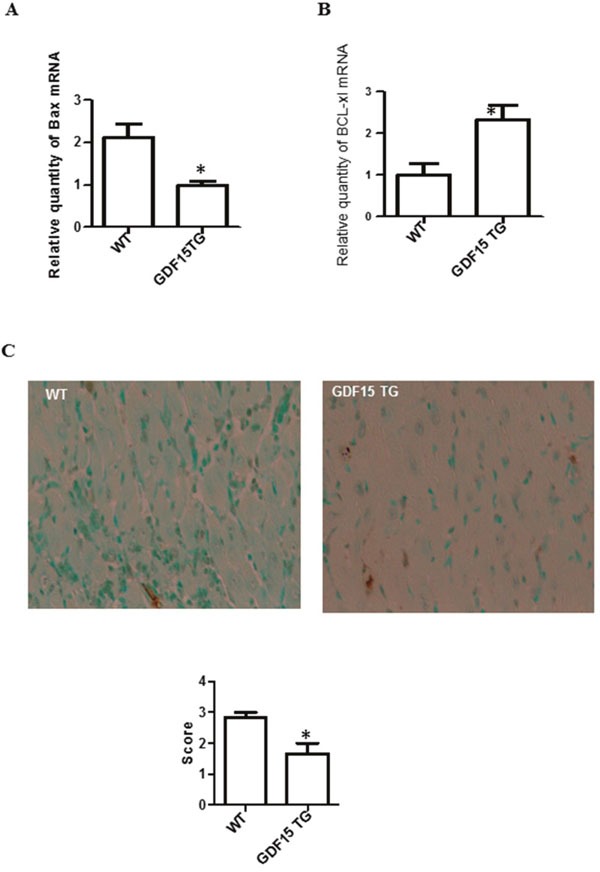
Over expression of GDF15 decreased the expression of pro- and anti-apoptotic genes and reduced apoptotic cells in heart grafts **(A & B)** Expression of Bax and Bcl-XL. RNA was extracted from the above heart graft tissues (Figure 1C–1F). The expression of Bax and Bcl-XL was determined using qRT-PCR. (A) Bax; (B) Expression of Bcl-XL. **(C)** Cell apoptosis detected by TUNEL assays. Heart grafts from Figure 1 were subjected to a TUNEL assays. Upper panel: representative images of TUNEL assays (n=6). Lower Panel: Semi score of apoptosis in WT and GDF15TG heart grafts. * p< 0.05 was defined as statistical significance.

I/R induces cell apoptosis is one of the mechanisms by which I/R causes organ damage. We found pro-apoptotic gene Bax was over expressed while anti-apoptotic gene Bcl-XL was decreased in I/R injury heart grafts compared with non-I/R injured tissues. This gene expression change was attenuated by over-expression of GDF15 (Figure 2A and 2B). We also used a TUNEL assay to detect cell apoptosis in the heart grafts. Figure 2C showed that there were significantly fewer apoptotic cells in GDF15 TG heart grafts than in wild type heart grafts.

**Figure 3:**
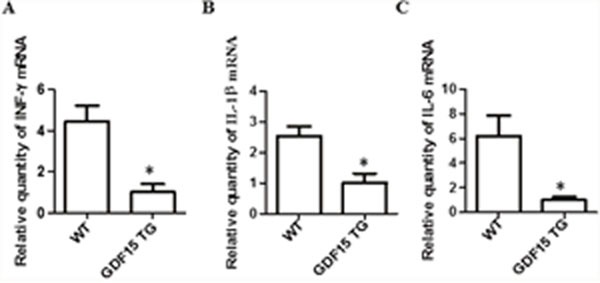
Inflammatory cytokine expression in heart grafts Heart grafts samples (n=4) were collected from grafts prepared by the procedure described by Figure 1C-F. Gene expression of inflammatory cytokines IFN-γ, IL-1β, and IL-6- was measured by qRT-PCR. GAPDH was used as an internal loading control gene. ΔΔCT was used to present relative changes of gene expression. * p< 0.05 was defined as statistical significance. **(A)** IFN-γ expression; **(B)** IL-1β expression; and **(C)** IL-6 expression.

It has been reported that myocardial cells in response to I/R injury up-regulated the expression of pro-inflammatory cytokines, for example, IFN-γ, IL-6, and IL-1 β in I/R injured hearts [19, 20]. Secreted cytokines recruit immune cells to move to the heart graft and produce proinflammatory cytokines, causing damage to grafts. GDF15 has been reported to act as an anti-inflammatory agent [21]. Accordingly, we detected and compared pro-inflammatory cytokine IFN-γ, IL-6, and IL-1 β expression in heart grafts by qRT-PCR. The expression of IFN-γ, IL-6, and IL-1 β was significantly decreased in the GDF15 TG heart grafts when compared with the wild type heart grafts (Figure 3A-3C). The expression of IL-6 and IL-1 β was confirmed at the protein level by immunohistochemistry (IHC) staining (Table 2). We also detected the expression of TNF-α by IHC. There was a reduction in the protein of TNF-a, but there was no significant difference between the two groups (Table 2).

**Table 2:** Semi-quantitative expression of proinflammatory cytokines detected by IHC

	IL-1β	IL-6	TNF-α
WT	2.75 ± 0.25	2.875 ± 0.125	2.250 ± 0.2500
GDF15 TG	0.9375 ± 0.0625	1.313 ± 0.2772	1.250 ± 0.2500
P value	0.0005	0.0209	0.0690

### GDF15 protects cardiomyocytes from cell apoptosis/death induced by I/R *in vitro*

**Figure 4:**
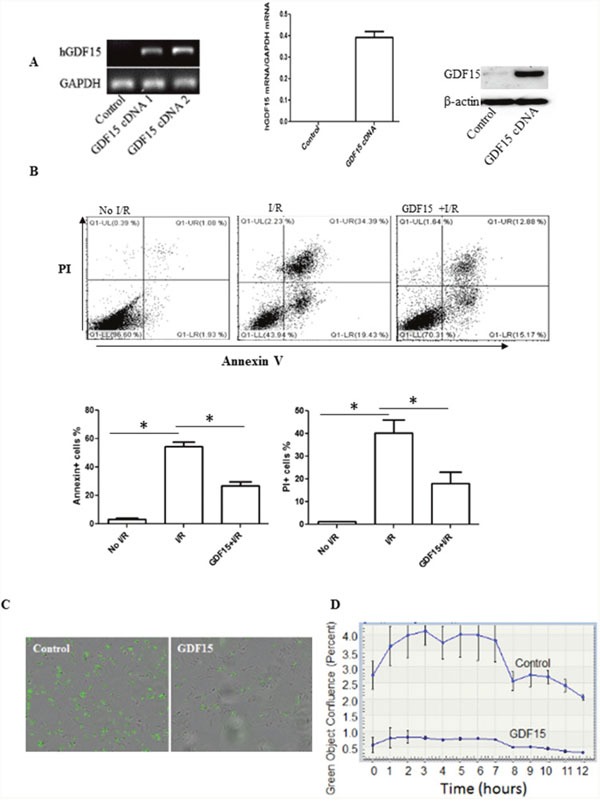
Over-expression of GDF15 prevents cell apoptosis and death induced by I/R *in vitro* H9C2 cells (80,000 cells/well) were plated in six well plates and allowed to culture at 37°C 5% CO_2_ overnight. Cells were infected with human GDF15 expression adenovirus or control null virus for 24h. Cells were then subjected to a hypoxia chamber with 0% O_2_ and 15% CO_2_ at 10°C for 16 h, followed by 24h reperfusion at 5% CO_2_, 28% O_2_ at 37°C. Gene expression of GDF15 was detected by RT-PCR and cell apoptosis was detected by double staining with FITC labeled Annexin-V and PI and flow cytometry. **(A)** Expression of human GDF15. Human GDF15 expression in H9C2 cells was detected by RT-PCR using primers specific to human GDF15 sequence and Western blotting. Left panel: regular RT-PCR; Middle panel: qRT-PCR. Right panel; Western blotting. **(B)** Cell apoptosis/death detected by Annenix-V and PI binding. Upper panel: representative data; bottom panel: Summarized data of flow cytometry for cell apoptosis/death. **(C)** Representative images of cell death detected by an incucyte system. **(D)** A cell death curve over time. Data are the summary of three independent experiments. * p< 0.05 was defined as statistical significance.

To further verify that the effect of GDF15 on cell death induced by cold I/R is protective, not causative, we cultured rat H9C2 cells, the most commonly used heart cell line for *in vitro* studies of I/R [22, 23] and treated them with GDF15 expression adenovirus prior to exposure to 16 h cold hypoxia at 10 oC followed by 24 h reperfusion at 37°C *in vitro*. We confirmed that GDF15 expression was up-regulated both at the mRNA and protein levels in GDF15 adenovirus infected cells (Figure 4A). To detect cell apoptosis and death, we double stained cells with Annexin-V-FITC and PI, and then conducted flow cytometry. As shown in Figure 4B, 34% of cells were Annexin-V^+^PI^+^ in the 16 h cold hypoxia /reperfusion group, while only 12% of cells were Annexin-V^+^PI^+^ in GDF15 adenovirus pretreated group. The data suggests that GDF15 has an anti-apoptotic function.

We also performed real-time quantitative live-cell analysis in an Incucyte using SYTOX Green nucleic acid stain (SYTOX) to confirm the effect of GDF15 on cell death in H9C2 cells. SYTOX is a green-fluorescent dye used to detect dead cells by binding to nucleic acid released from dead cells. We cultured and infected H9C2 cells with GDF15 expression adenovirus and subjected them to 16 h cold ischemia in an ischemic chamber. After taking the cells out of the ischemic chamber, we replaced the cell medium with normal culture medium with 10% FBS. We immediately added SYTOX Green at a final concentration of 0.5μmol/L to the culture and placed the cell plates into an Incucyte system. Cells were scanned for 24 h. As shown in Figure 4C, there were more green fluorescent dead cells in the wells treated with control null adenovirus at 3h after an addition of SYTOX. In contrast, there were much fewer green fluorescent dead cells in the cell wells treated with GDF15 expression adenovirus. Figure 4D is a plot of green object confluence over scanning time, and clearly shows that I/R induced cell death was attenuated by over-expression of GDF15.

### GDF15 protects heart cells from I/R injury through the Foxo3a signaling pathway

**Figure 5:**
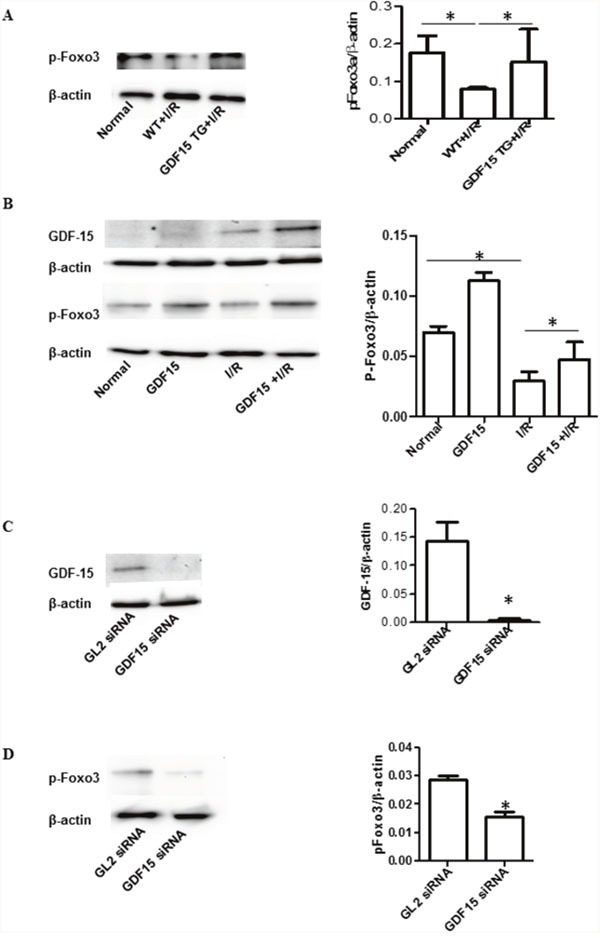
GDF15 increased phosphorylation of Foxo3a **(A)** Phosphorylation of Foxo3a in the heart grafts. Total protein was extracted from heart grafts at day 3 post transplantation (n=6). Phosphorylation of Foxo3a was detected by Western blotting with primary antibodies against phosphorylated Foxo3a. Left panel: Representative images of Western blotting for Foxo3a. Right panel: Densitometry values of p-Foxo3a/β-actin. **(B)** Over expression of GDF15 recovered expression of phosphorylated Foxo3a *in vitro*. H9C2 cells (80,000 cells/well) were plated in six well plates and allowed to culture at 37°C 5% CO_2_ overnight. Cells were infected with human GDF15 expression adenovirus or control null virus, for 24 h, Cells were then subjected to a hypoxia chamber with 0% O_2_ and 15% CO_2_ at 10°C for 16 h, followed by a 24 h reperfusion period at 5% CO_2_, 28% O_2_ at 37°C. Total protein was extracted from the cells and the expression of GDF15, p-Foxo3a and β-actin were detected by Western blotting using appropriate antibodies. Left panel: Representative images of Western blotting for p-Foxo3a, and β-actin from three independent experiments. Right panel Densitometry values of p-Foxo3a/β-actin. **(C)** GDF15 siRNA reduced GDF15 expression. H9C2 cells were transfected with GDF15 siRNA prior to hypoxia/reperfusion. Left panel: Representative images of Western blotting for GDF15 and β-actin from three independent experiments. Right panel Densitometry values of GDF15/β-actin. **(D)** GDF15 siRNA reduced phosphorylated Foxo3a expression. Left panel: Representative images of Western blotting for p-Foxo3a, and β-actin from three independent experiments. Right panel Densitometry values of p-Foxo3a/β-actin* p< 0.05 was defined as statistical significance.

It has been reported that GDF15 prevents I/R injury through activation of PI3K and AKT signaling [13]. This signaling was confirmed by this study (data not shown). In order to explore other possible underlying signaling pathways, we detected Foxo3a activation in heart grafts in wild type and GDF15 TG mice as Foxo3a has been reported to be involved in renal and liver I/R injury [24–26]. As shown in Figure 5A, I/R decreased Foxo3a phosphorylation in the wild type grafts as compared with the heart without I/R injury, whereas the expression of p-Foxo3a in the GDF15 TG I/R injured grafts was significantly increased as compared with the wild type I/R injured grafts and was almost recovered to the levels seen in hearts without I/R injury. These data indicate that GDF15 promotes phosphorylation of Foxo3a.

To further validate the involvement of Foxo3a activation, we performed an *in vitro* experiment using the H9C2 cell line. We cultured and treated H9C2 cells with ahuman GDF15 expressing adenovirus prior to subjecting them to a cold hypoxia /reperfusion environment. As shown in Figure 5B, we found that *in vitro* cold hypoxia /reperfusion also reduced Foxo3a phosphorylation as compared with the control cells cultured in normal cell culture conditions and that treatment with human GDF15 expression adenovirus (GDF15 cDNA) increased p-Foxo3a. More interestingly, pre-infecting cells with GDF15-adenovirus prior to exposure to a cold hypoxia environment prevented the reduction of p-phosphorylated Foxo3a expression (Figure 5B).

Furthermore, we transfected H9C2 cells with GDF15 siRNA for 24 h and then exposed these transfected cells to a cold I/R environment. After the 24 h reperfusion period, we detected expression of GDF15 and p-phosphorylated Foxo3a. GDF15 siRNA significantly down-regulated the expression of GDF15 (Figure 5C) and also decreased p-Foxo3a expression (Figure 5D). GDF15 siRNA also increased cell apoptosis/death (data not shown). The data further demonstrated that the effect of GDF15 on preventing cell death against I/R is associated with activation of Foxo3a signaling.

**Figure 6:**
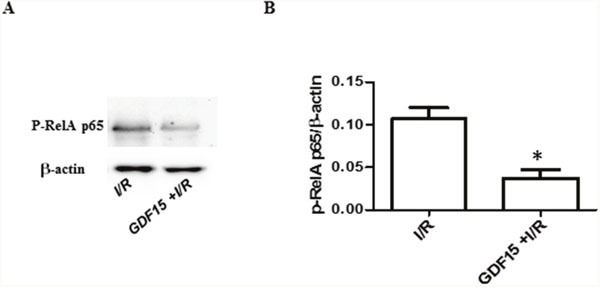
The expression of p-RelA p65 Cells were treated and protein was extracted from the cells as Figure 5. The expression of p-RelA p65 was detected by Western blotting. **(A)** Representative image from three independent experiments. **(B)** Densitometry values for p-RelA p65/β-actin. * p< 0.05 was defined as statistical significance.

It has been reported that the NFκB signaling pathway is activated during I/R which leads to inflammatory response [27]. The Rel A p65 subunit involved in this pathway is up-regulated in injured hearts [28] and the depletion of p65 protects the injured heart [29]. To determine whether GDF15 protective effect on I/R injury is through inhibition of the NFκB signaling pathway, we detected phosphorylation of Rel A p65 by Western blotting. The result showed that over-expression of GDF15 reduced the phosphorylation of Rel A p65 (Figure 6), suggesting that GDF15 prevents the activation of the NFκB signaling pathway.

## DISCUSSION

I/R injury occurring during the heart transplantation process remains a major factor in graft dysfunction and chronic rejection. In this study, we demonstrated that up-regulation of GDF15 in heart grafts is protective in response to cold I/R injury in heart transplantation and that over expression of GDF15 can protect donor hearts from cold I/R injury through inhibition of inflammation and apoptosis. Furthermore, we demonstrated that an underlying mechanism of GDF15 cardio protection is the inhibition of the Foxo3a signaling and NFκB signaling pathways.

GDF15 is an immediate early gene that acts in response to stresses and is rapidly up-regulated in order to reduce and/or prevent damage. In a murine warm I/R injury model induced by coronary artery ligation, GDF15 has been demonstrated to protect the heart from I/R injury through inhibition of leukocyte integrin activation in response to permanent and transient myocardial infarction [30]. The ability of GDF15 to inhibit neutrophil infiltration in an inflammatory-like response to I/R has been suggested [16]. In this study, we found that neutrophil infiltration occurring in a heart transplant setting, with cold I/R, was decreased with the overexpression of GDF15. Our study also showed that pro-inflammatory cytokine (IFN-γ, IL-6, IL-1β and TNF-α) expression was impeded by the overexpression of GDF15, in a cold I/R model. Furthermore, we observed that over-expression of GDF15 inhibited the phosphorylation of Rel A p65, a member of the NFκB family. Our data suggest that the attenuation of inflammation by GDF15 is mediated by the inhibition of the NFκB signaling pathway. This finding is aligned with a report on prostatic inflammation in which over expression of GDF15 in prostatic cancer cells led to decreased NFκB-mediated inflammation [31]. Overall, our study shows a new circumstance in which GDF15 protects against inflammation, and further supports GDF15 as a cardio protective agent against inflammation under ischemic conditions.

Cell apoptosis is one of the manifestations of I/R injury in organ transplantation [32]. GDF15 expression has been linked to either anti- or pro-apoptotic function in several disease models. It is conceivable that GDF15 plays a pro-apoptotic protein role in cancer cells, whereas GDF15 acts as an anti-apoptotic protein in non-cancer cells [14, 33, 34]. The latter was described by Kempf and colleagues, who discovered GDF15 induction in the heart as a defense mechanism from warm I/R injury by reducing apoptosis in a coronary artery ligation model [13]. Later, Heger and colleagues [35] further identified GDF15 as an anti-apoptotic protein in ventricular cardiomyocytes from male Wistar rats, by showing that the effects of three different apoptotic inducers (angiotensin II, NO, and TGF-β 1) could be attenuated by the presence of GDF15. In this study, we found that the number of apoptotic cells was decreased in GDF15 TG grafts as detected by TUNEL assays. The expression of apoptotic genes Bax were also reduced in heart grafts with overexpression of GDF15, compared to WT grafts. Additionally, our *in vitro* result with H9C2 cells showed that, over-expression of GDF15 reduced H9C2 cell apoptosis under cold hypoxia/reperfusion stress. Taken together, GDF15 is an anti-apoptotic protein that protects heart cells against apoptosis induced by cold I/R in a heart transplant model. The ability of GDF15 to prevent cell apoptosis suggests that GDF15 has potential therapeutic value in preventing I/R in heart transplantation.

More importantly, this is the first study showing that Foxo3a is a downstream protein of GDF15 and that its regulation through GDF15 confers cardiac protection from cold I/R injury in transplantation. Foxo3a is a member of the forkhead transcription factor family and has been studied for its role as a tumor suppressor due to its pro-apoptotic function [36, 37] and for an association with human longevity [38]. It has been reported that Foxo3a influences hypertrophy of heart cells, in the absence of active Akt [39] and promotes cardiomyocyte survival under oxidative stress through reduction of reactive oxygen species and cell death [40]. More recent studies have shown that Foxo3a signaling is involved in kidney and liver I/R injury *in vivo* [24, 25] and I/R injured cardiomyocytes and cardio endothelial cells *in vitro* [26, 41]. Foxo3a is also associated with inflammation and oxidative stress [42]. Our study showed that cold I/R decreased the phosphorylation of Foxo3a in the WT grafts, as compared to the control heart grafts without 24h cold I/R. We also showed that the decrease in phosphorylation of Foxo3a can be reversed by over-expression of GDF15. This result indicates a direct effect of GDF15 on Foxo3a and possible mechanism for GDF15′s protective function on apoptosis and inflammation, which has not been previously demonstrated. Our *in vitro* study on H9C2 cells also showed that overexpression of human GDF15 by infecting these cells with a human GDF15 expressing adenovirus led to increased phosphorylation of Foxo3a in cold I/R conditions. By contrast, silencing GDF15 with siRNA decreased the expression of phosphorylated Foxo3a and increased cell death, demonstrating a direct relationship between the expression of GDF15 and Foxo3a activation. Combined, our data indicate that GDF15 prevents apoptosis and inflammation, thus protects heart grafts from I/R injury through regulation of Foxo3a signaling.

Recent studies have shown that there is a link between Foxo3 and NFκB signaling, both of which play important role in anti-apoptosis and oxidative stess [27, 43]. In an aging related study, Salminen et al reported that Foxo signaling acted as an antagonist of NFκB [44]. However, a study by Li et al [45] reported that Foxo3a is a positive regulator of NFκB and over-expression of Foxo3a increased the nucleus translocation of p65 in cancer cells. The relationship between Foxo and NFκB signaling is controversial. In this study, we found over expression of GDF15 increased Foxo3 phosphorylation while reduced activation of NFκB signaling. These data imply that there might be a crosstalk between Foxo3 and NFκB signaling and Foxo3 acts as antagonist of NFκB signaling. The protection of GDF15 might be through either regulating Foxo3 which subsequently inhibits NFκB signaling, or separately regulating these two signaling pathways, which needs to be verified in future study. Nonetheless, our study is the first to demonstrate that GDF15 regulates Foxo3 signaling.

In addition to the anti- apoptosis and anti-inflammation function of GDF15, previous cancer studies as well as our unpublished data show that GDF15 has an immunosuppressive function on antigen presenting cells such as dendritic cells and T cells, which in turn might suppress alloimmune response against allografts in organ transplantation. Our unpublished results also showed that over expression of GDF15 in recipients can delay allograft immune rejection in a major histocompatibility complex (MHC) full mismatch heart transplantation model. Therefore, over-expression of GDF15 in a donor heart may not only prevent donor hearts from cold I/R injury in heart transplantation, but it could also reduce immunogenicity, leading to prolongation of allograft survival.

In conclusion, this study first demonstrates that GDF15 is a promising target for preventing cold I/R injury in heart transplantation and shows the association of GDF15 with Foxo3a. We also demonstrate that the protective effect of GDF15 is mediated by Foxo3 and NFκB signaling.

## MATERIALS AND METHODS

### Animals

C57B/6 WT mice were purchased from Charles River Laboratories (Canada). GDF15 Transgenic (TG) mice ubiquitously expressing high levels of human GDF15 (hNAG1) under the control of the chicken *β*-actin promoter (CAG) C57BL/6 mice were kindly provided by Dr. Baek (University of Tennessee). All experiments in the study were performed in accordance with the guidelines established by the Canadian Council of Animal Care and were approved by the Animal Care Committee of the University of Western Ontario.

### Cell line and cell culture

Rat heart cell line H9C2 were purchased from ATCC (Manassas, VA 20108), cultured and maintained in DMEM medium (Invitrogen, Canada) which were supplemented with 10% fetal bovine serum (Sigma, Oakville, ON, Canada) and 100 U penicillin and streptomycin.

### Heterotopic heart transplantation with prolonged ischemia reperfusion

A syngeneic murine heterotopic heart transplantation was performed as described previously [17]. C57BL/6 male mice and GDF15 TG male mice at 8 weeks of age were anesthetized with Ketamine/Xylene and the hearts were excised, and preserved in UW solution for 24h at 4°C. 24h later, the preserved hearts were then implanted into the same strain recipient mice as the donors. On day 3 post transplantation, transplant mice were sacrificed and heart grafts were taken for histopathological examination, gene expression and fibrosis detection.

### *In vitro* ischemia reperfusion model

H9C2 cells were plated in a 6 well plate (80,000 cells/well) and cultured in DMEM medium supplemented with 10% fetal bovine serum and 100 U/ml penicillin and streptomycin overnight. DMEM culture medium was replaced by deoxygenized PBS and then placed in an *in vivo* 2 hypoxia workstation (Baker Ruskinn, Sanford, MA) with 0% O2 at 10 oC for 16 h. After 24h of the hypoxia treatment, PBS was removed and new complete culture medium was added to the cells. Cells were moved to a normal culture environment with 5% CO_2_ and 28% O_2_ at 37°C for 24 h.

### siRNA transfection and adenovirus infection

H9C2 cells (80,000/well) were plated in a six well plate and cultured overnight and then transfected with 1μg mouse GDF15 siRNA using 2μl lipofectamine 2000 (Life technologies, Burlington, ON, Canada) in 600 μl opti-medium as instructed by the manufacturer. 4h after transfection, 600 μl culture medium containing 20% FBS was added to the transfected cells and the cells continued to be cultured for overnight.

For infection with an adenovirus, H9C2 cells (80,000/well) were plated in a six well plate and cultured for overnight and then infected with human GDF15 cDNA expression adenovirus (SignaGen Laboratories, Rockville, MD) at the titer of 100 Multiplicity of Infection (MOI) in a 600 μl FBS-free DMEM for 6 h. Then, 600 μl culture medium containing 20% FBS was added to the infected cells and the cells were cultured overnight.

### Reverse transcriptase–polymerase chain reaction (RT-PCR) and quantitative PCR (qPCR)

Total RNA was extracted from cells and heart tissues using Trizol (Qiagen, Toronto, Ontari, Canada). 3 μg of total RNA was used to synthesize cDNA using reverse transcriptase (New England Biotechnologies, Ipswich, MA). Gene expression of GDF15, IL-6, IL-1β, Bax, Bcl-XL, and GAPDH were detected by qPCR using the SybGreen method. Primer sequences used for PCR were listed: GDF15 (mouse and rat) F: 5′-ttctgtggggacggtcag-3′and R: 5′-cgggtgaccaggctaattc-3′), human GDF15 (hNAG-1): F: 5′-ctccagattccgagagttgc-3′ and R 5′- agagatacgcaggtgcaggt-3′; IL-6, 5′-ccggagaggagacttcacag-3′(F) and 5′-ggaaattggggtaggaagga-3′(R); IL-1β, 5′-caggcaggcagtatcactca-3′(F) and 5′-tgtcctcatc ctggaaggtc-3′(R); Bax, 5′- aggcctcctctcctacttcg (F) and 5′-aaatgcctttccccttcccc-3′(R); Bcl-XL, 5′- cctcctccccgacctatgat -3′(F), 5′-cccggttgctctgagacatt-3′(R), and GAPDH, 5′-caggagcgagaccccactaacat-3′(F), 5′-gtcagatccacgacggacacatt-3′(R).

qPCR was conducted in a 10 μl PCR with 1 x SybrGreen mixture (Bio-Rad), 100 nM primers, and 1 μl of cDNA, with the following thermal profiling: an initial activation step was carried with at 95°C for 2 mins, followed by 40 cycles of: 95°C for 15 s, 60°C for 30 s, and 72°C for 20 s.

Expression levels between I/R and non-I/R were quantitatively compared using the ΔΔCt method with GAPDH as the endogenous control for RNA expression.

### Western blotting

H9C2 cells were collected and washed with PBS. Total proteins were extracted with RIPA buffer containing protease inhibitor MSCF, followed by 3 cycles of 5 second sonication. For heart tissue, 10 mg of heart tissue was homogenized with PRIPA buffer containing protease inhibitor MSCF using a manual homogenizer on ice prior to sonication. Cell lysate and tissue lysate were centrifuged for 20 min at 15,000 rpm and supernatant was collected. The concentration of protein was measured using the Bradford method with Bradford 1x Dye reagent (BioRad, Mississauga, Ontario, Canada). 25μg total protein was loaded on 12% polyacrylamide gel and run for 60 min-90 min at 100 volts. Separated proteins were transferred to a PVDF membrane. Transferred membranes were blocked with 5% fat-free milk in TBST for 30 min at room temperature and then blotted with the primary antibodies against GDF15 (1:1000 dilution, Sigma), phosphorylated Foxo3a (1:1000 dilution, Cell Signaling Technology, Danvers, MA), total Foxo3a (1:2000 dilution, Cell Signaling Technology), phosphorylated RelA p65 (1:2000 dilution, Cell Signaling Technology, Danvers, MA), total RelA p65 (1:2000 dilution, Cell Signaling Technology), and β-actin (1:4000 dilution, Santa Cruz Biotechnologies, San Diego, CA) at 4°C for overnight. The blotted membranes were washed with TBST containing 0.25% Tween-20 for 10 min at room temperature and repeatedly washed for three times. Washed membranes were blotted with appropriate secondary antibodies (Santa Cruz Biotechnologies) for 30 min at room temperature. Proteins were developed with ECL kits (Bio-Rad, Hercules, CA 94547) and visualized with a FluorChem M system (ProteinSimple, San Jose, CA).

### Histological analysis

Heart grafts were collected from mice and tissue slices were fixed in 10% formalin and processed for histological examination using standard techniques. Formalin tissue was embedded in paraffin and 5 μm sections were stained with hematoxylin and eosin stain (H&E). Histological changes of heart grafts were assessed on damage of the epicardium, myocardium and endocardium by a pathologist in a double blind method. Injury of the epicardium, myocardium and endocardium, infarction, neutrophil infiltration and fibrosis were scored using a five-point scoring system based on injury area of involvement as follows: 0, <10%; 1, 10% to 25%; 2, 25% to 50%; 3, 50% to 75%; and 4, 75% to 100%.

### Terminal deoxynucleotidyl transferase-mediated dUTP nick-end labelling (TUNEL) assay

Cell apoptosis in heart grafts was detected by the TUNEL assay using paraffin embedded tissue sections and an *in situ* cell death detection kit according to the manufacturer's instructions (Roche, Mississauga, ON). Apoptosis was semi-quantitatively assessed using a five point scoring system based on apoptotic area of involvement as follows: 0, <10%; 1, 10% to 25%; 2, 25% to 50%; 3, 50% to 75%; and 4, 75% to 100%.

### Myeloperoxidase (MPO) activity

Neutrophil infiltration in heart tissue was examined by detection of MPO activity. Immunohistochemistry was performed using the standard protocol. Paraffin-embedded tissue sections were deparaffinised, rehydrated, blocked and incubated with a polyclonal rabbit MPO antibody (1:100, NeoMarkers, Fremont, CA). This was followed by incubation with EnVision+ anti-rabbit-HRP (Dako, Carpinteria, CA). Sections were then incubated with the chromogenic substrate and counterstained with hemotoxylin.

### Immunohistochemistry staining and semi-quantitative analysis

Fresh frozen heart tissues were embedded with OCT and 5μm slides were sectioned. Heart tissue sections were stained with primary antibodies including IL-6, IL-1β, and TNF-α Ab (Santa Cruz Biotechnologies), at the manufacturer's suggested dilution, followed by staining with fluorescent secondary antibodies: FITC labeled goat, anti-rabbit and CruzFluor™ 488 labeled goat anti mouse secondary antibodies. Images were taken under a fluorescent microscope at 200× magnification.

Expression of inflammatory cytokines detected by IHC was semi-quantified using a five point scoring based on injury area of involvement as follows: 0, <10%; 1, 10% to 25%; 2, 25% to 50%; 3, 50% to 75%; and 4, 75% to 100%.

### Trichrome staining

Fibrosis was detected by Trichrome stain using a Trichrome stain kit (Abcam Inc, Toronto, Canada) following the manufacturer‘s instruction.

### Real-time quantitative cell death analysis using an Incucyte system

To quantitatively detect cell death in real time; we used an Incucyte system (Essen BioScience Ann Arbor, MI) and SYTOX Green Nucleic Acid Stain (Thermofisher, Mississauga, Canada). H9C2 cells were cultured in a 24-well plate and treated according to experimental requirements as described in the results section. SYTOX Green (a nucleic acid staining reagent) was added to the cell culture with a final concentration of 0.5μmol/L. Cell plates were immediately placed into an Incucyte system. Cells were scanned every hour for 24h with an Excitation/Emission at 504/523 nm. Dead cells were visualized as green fluorescent cells based on the incorporation of SYTOX Green Nucleic Acid Stain into the nuclei and chromosomes.

### Annexin V/propidium iodide (PI) staining

Cell apoptosis of H9C2 cells was assayed using an FITC-labeled Annexin V (Annexin V-FITC) apoptosis detection kit (BD Biosciences). Cells were collected after the indicated treatment, washed twice with cold PBS and then suspended in 1×binding buffer, followed by staining with Annexin V-FITC and PI in the dark. Immediately, the percentage of apoptotic cells was quantified by flow cytometry (FACSCalibur, BD Biosciences) according to the manufacturer's instructions.

### Statistical analysis

Data are presented as mean ± SEM. Data were analyzed by Graphpad Prism. A one-way analysis of variance (ANOVA) was applied for comparison among three groups with the Newman-Keuls test. The Student's t-test was applied for comparisons between two groups. Differences were considered statistically significant when P value <0.05.

## Abbreviations

I/R: Ischemia reperfusion injury
GDF15: Growth differentiation factor 15
GDF15 transgenic mice: GDF15 TG
NAG: Anti-inflammatory drug-activated gene)
TGF-β: Transforming growth factor beta
Foxo3a: Forkhead box O3a
NFκB: Nuclear factor kappa B
Bcl-xL: B-cell lymphoma-extra large
BAX: BCL2 Associated X
IFN-γ: Interferon gamma
IL-6: Interleukin 6
IL-1 β: Interleukin 1 beta
IHC: immunohistochemistry
TNFα: Tumor necrosis factor
FITC: Fluorescein isothiocyanate
PI: Propidium iodide
SYTOX: Green nucleic acid stain
FBS: Fetal bovine serum
siRNA: Small interference RNA
